# Anti-adherent effects of *Rhizophora apiculata* bark and leaf extracts and computational prediction of the effects of its compound on β-tubulin interaction in *Acanthamoeba triangularis* genotype 4

**DOI:** 10.14202/vetworld.2024.2829-2845

**Published:** 2024-12-18

**Authors:** Siriphorn Chimplee, Imran Sama-ae, Suthinee Sangkanu, Watcharapong Mitsuwan, Julalak Chuprom, Rachasak Boonhok, Dhrubo Ahmed Khan, Partha Biswas, Md Nazmul Hasan, Hazel Anne Tabo, Cristina C. Salibay, Polrat Wilairatana, Maria L. Pereira, Muhammad Nawaz, Ragini Bodade, Shanmuga S. Sundar, Alok K. Paul, Veeranoot Nissapatorn

**Affiliations:** 1 General Education Department, School of Languages and General Education, Walailak University, Nakhon Si Thammarat, 80160, Thailand; 2 Department of Medical Technology, School of Allied Health Sciences and Center of Excellence Research for Melioidosis and Microorganisms (CERMM), Walailak University, Nakhon Si Thammarat, 80160, Thailand; 3 School of Allied Health Sciences, Southeast Asia Water Team (SEA Water Team) and World Union for Herbal Drug Discovery (WUHeDD), Walailak University, Nakhon Si Thammarat, 80160, Thailand; 4 Akkhraratchakumari Veterinary College, Walailak University, Nakhon Si Thammarat, 80160, Thailand; 5 Laboratory of Pharmaceutical Biotechnology and Bioinformatics, Department of Genetic Engineering and Biotechnology, Jashore University of Science and Technology, Jashore-7408, Bangladesh; 6 ABEx Bio-Research Center, East Azampur, Dhaka-1230, Bangladesh; 7 Department of Biological Sciences, College of Science, De La Salle University-Dasmarinas, Cavite, Philippines; 8 Department of Clinical Tropical Medicine, Faculty of Tropical Medicine, Mahidol University, Bangkok, Thailand; 9 Department of Medical Sciences and CICECO-Aveiro Institute of Materials, University of Aveiro, Aveiro, Portugal; 10 Department of Nano-Medicine Research, Institute for Research and Medical Consultations, Imam Abdulrahman Bin Faisal University, Dammam, Saudi Arabia; 11 Life Science Division, Institute of Advanced Study in Science and Technology (IASST), Vigyan Path, Paschim Boragaon, Garchuk, Guwahati, Assam, India; 12 Department of Biotechnology, Aarupadai Veedu Institute of Technology, Vinayaka Mission’s Research Foundation, Paiyanoor, Chennai, Tamil Nadu, India; 13 School of Pharmacy and Pharmacology, University of Tasmania, Hobart, TAS 7001, Australia

**Keywords:** *Acanthamoeba triangularis*, anti-adherent activity, molecular docking, molecular dynamic simulation, *Rhizophora*, tubulin

## Abstract

**Background and Aim:**

*Acanthamoeba*, an opportunistic protozoan, exists widely in natural sources and can cause infections in humans and animals. The absence of effective monotherapy after the initial infection leads to chronic disease and recurrence. Tubulin protein is a vital target for design-targeted drug discovery. Anti-tubulin drugs are also used to treat *Acanthamoeba* infections, although resistance to these drugs has been observed. Therefore, it is necessary to identify a new targeted drug for *Acanthamoeba* infections. Therefore, this study aimed to assess the *in vitro* activity of ethanol extracts of *Rhizophora apiculata* extracts (RAE) against *Acanthamoeba* spp. and to predict its chemical compound on β-tubulin interaction.

**Materials and Methods:**

In this study, anti-*Acanthamoeba* activity with minimal inhibitory concentration (MIC) and minimal parasiticidal concentration (MPC) determination of ethanolic RAE from leaves, blossoms, buds, branches, and barks was tested on four *Acanthamoeba* trophozoites and cysts: *Acanthamoeba triangularis* WU 19001, *Acanthamoeba polyphaga* American Type Culture Collection (ATCC) 30461, *Acanthamoeba castellanii* ATCC 50739, and *A. castellanii* ATCC 30010. The inhibitory effect on adherence was determined by the ability of *Acanthamoeba* adherence on 96-well plates, and its adhesive acanthopodia structure was evaluated using scanning electron microscopy analysis. In addition, the minimum cytotoxic concentrations (MCC) of *R. apiculata* leaf extract (RALE) and bark extract (RABE) were evaluated on Vero and HaCaT cell lines using the MTT assay. Phytochemical compounds from RALE and RABE were also analyzed by gas chromatography-mass spectrometry (GC-MS). Molecular docking and molecular dynamic analysis predicted the binding sites of chemicals in extracts and β-tubulin protein.

**Results:**

The results revealed that *A*. *triangularis* and *A. polyphaga* trophozoites had the highest inhibition at 90% at a MIC of 8 mg/mL after treatment with RALE and RABE, respectively, at 24 h. Those MPC values were exhibited at 16 mg/mL against *A*. *triangularis* trophozoites. In addition, both extracts inhibited the adhesive properties of all *Acanthamoeba* approximately 80%–90% at 4 mg/mL, as well as adherent structural acanthopodia loss. MCC was 0.25 mg/mL, provided to be harmless to mammalian cells. GC-MS analysis supported that 8 and 11 major phytochemicals were from RABE and RALE, respectively. Molecular docking and molecular dynamics demonstrated that *Acanthamoeba*-β-tubulin exhibited potent root-mean-square deviation, root mean square fluctuation, and binding free energy values with clionasterol (from RABE and RALE) and stigmasterol (from RALE). Based on our results, ethanolic RABE and RALE exhibited anti-*Acanthamoeba* activity in reducing adhesion. *In silico* showed that promising clionasterol and stigmasterol interacted with a targeting β-tubulin.

**Conclusion:**

The RABE and RALE exhibited a potential anti-adherent effect on *A. triangularis*, low toxicity, and the clionasterol and stigmasterol in RABE and RALE predicted to interact the targeted β-tubulin. These agents may be used as alternative therapeutic agents in the management of disease using a sustainable one-heath approach.

## Introduction

*Acanthamoeba* are normally free-living pathogens that are ubiquitous in the environment. The trophozoites and cysts have two morphologically distinct life cycle stages. The cyst stage is roughly spherical and sessile, but trophozoites have an amoeboid characteristic and are motile [1]. *Acanthamoeba* can cause granulomatous encephalitis and blinding keratitis [2]. Although there are various chemotherapeutic treatments for *Acanthamoeba* infections, most are laborious and only partially effective [2]. Biguanides, chlorhexidine (CHX), polyhexamethylene (PHMB), and diamidine (propamidine and hexamidine) are the first-choice treatments for *Acanthamoeba* keratitis (AK) [3, 4]. This combination is effective against trophozoites and cysts, but can cause ocular toxicity and increased cystic resistance, even at low concentrations [3–6]. In addition, granulomatous amebic encephalitis, which is most frequently related to immunosuppression, is typically fatal despite some progress in the treatment of *Acanthamoeba* [7]. In addition to human corneal infection, there is evidence of a possible connection between *Acanthamoeba* and keratitis in animals, especially in dogs [8, 9]. Awareness of veterinary professionals of the potential threat associated with AK and more studies on the extent of its occurrence and clinical impact in the field are of paramount [8]. Topical treatments, including CHX, PHMB, and dexamethasone, and systematic therapy in AK animals remain unsuccessful when the disease becomes more severe [8, 9]. Therefore, there is a need for the development of novel, more effective treatments for *Acanthamoeba* infections.

Tubulin is a crucial structural component of the cytoskeleton of eukaryotic cells. It is essential for organelle movement, cellular motility, and chromosomal segregation [10]. Tubulins are substantially conserved across all phyla; however, organisms exhibit a range of susceptibility and resistance to various antimicrotubular chemical classes [11]. *Acanthamoeba* spp. has been studied for having different α- and β-tubulins [11]. In addition, *Acanthamoeba castellanii* and *Acanthamoeba polyphaga* are resistant to oryzalin, paclitaxel, vinblastine, albendazole, and colchicine, which are known to target tubulin protein [11]. There is scant data on anti-*Acanthamoeba* activity in tubulin proteins; consequently, our study proposed to investigate the selected targeting protein of *Acanthamoeba* with potentially active compounds from *Rhizophora* extracts.

*Rhizophora apiculata*, also known as Asiatic mangrove, is a member of the Rhizophoraceae family that is extensively distributed throughout the tropical and subtropical coastal regions. Conventionally, mangroves have been used as pesticides, insecticides, and medicinal plants. It contains bioactive compounds such as steroids, phenolic acids, flavonoids, and terpenoids [12], which have a wide range of pharmacological activities, including anti-fungal, anti-bacterial, anti-septic, anti-inflammatory, anti-ulcer, and antioxidant activities [13, 14]. Recently, the anti-parasitic activity of the crude extract of *Rhizophora mucronata* bark was reported to have anti-intestinal protozoan activities against *Entamoeba histolytica* and *Giardia intestinalis*, with minimal inhibitory concentration (MIC) values of >1 and 0.5 mg/mL, respectively [15]. So far, little is known about the anti-*Acanthamoeba* activity of this Asiatic mangrove plant and its targeting of tubulin protein.

Therefore, this study aimed to assess the *in vitro* activity of *R. apiculata* extracts (RAE) against *Acanthamoeba* spp. Our study further evaluated the binding sites of selected phytochemicals in these extracts with tubulin protein using an *in-silico* study including molecular docking and dynamic simulation.

## Materials and Methods

### Ethical approval

The study was approved by the Committee of the Biosafety Guidelines for Scientific Research of Walailak University, Nakhon Si Thammarat, Thailand (Ref. No. WU-IBC-66-020).

### Study period and location

The data collection for this study was conducted from March 2022 to March 2023 at Tropical Medicine Laboratory, Research Institute for Health Sciences (RIHS), and Center for Scientific and Technological Equipment, Walailak University, Nakhon Si Thammarat, Thailand.

### Plant material and extraction

**Figure-1:**
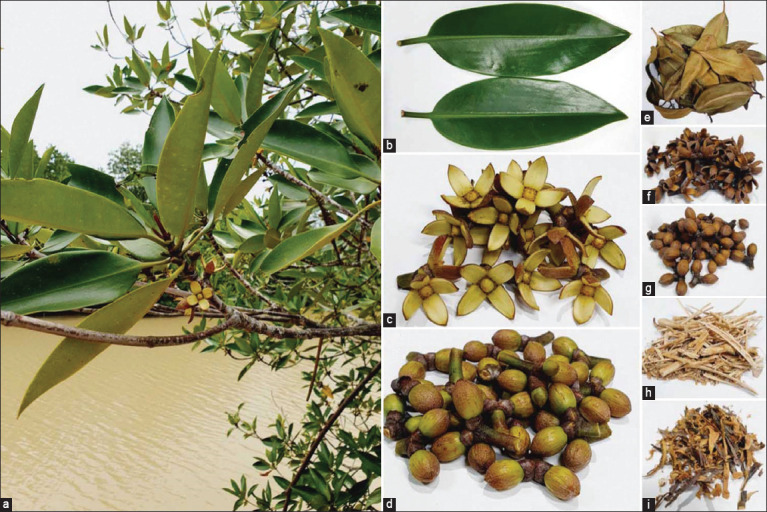
*Rhizophora apiculata* and its parts used in this study. Raw materials: (a) branch, (b) leaf, (c) blossom, and (d) bud. Dried samples: (e) leaf, (f) blossom, (g) bud, (h) branch, and (i) bark.

*R. apiculata* was collected from a mangrove forest in Tha Sala, Nakhon Si Thammarat, Thailand. This plant was identified by Assistant Professor Dr. Jariya Sakayaroj, Head of the Biology Division, School of Sciences, Walailak University (Figure-1a). The identification of this selected plant is based on the morphological characteristics of its leaves (size; small and the range is 4–8 × 7–18 cm and shape; lance), as appropriate. No specific permission is required for this location. Plant parts were used in this study, such as leaves (Figure-1b), blossoms (Figure-1c), buds (Figure-1d), branches (Figure-1h), and bark (Figure-1i). Plant parts were dried (Figures-1e-i) and then ground in a mortar. The dried plant was soaked in 95% ethanol (1:3) for 7 days. The solution was filtered using filter paper (GE Healthcare Life Sciences, IL, USA). The filtrate was then evaporated using a rotary vacuum evaporator (Hei-VAP Advantage HL/G3; Heidolph, Germany) to obtain the extracts. The extracts were dissolved in 99.5% dimethyl sulfoxide (DMSO; RCI Labscan, Bangkok, Thailand) to obtain 400 mg/mL stock solution and stored at −20°C until use.

### *Acanthamoeba* spp. cultivation

Four strains of *Acanthamoeba* were tested; namely, *Acanthamoeba triangularis* WU19001, an environmental strain, was isolated from Walailak University [16], while *A. polyphaga* American Type Culture Collection (ATCC) 30461, *A. castellanii*, a non-pathogenic strain (ATCC 30010), and *A. castellanii* pathogenic strain (ATCC 50739) were purchased from the ATCC (Manassas, VA, USA). *Acanthamoeba* culture was performed using the following procedure [16, 17]. In brief, trophozoites were grown in Peptone Yeast Glucose (PYG) Broth medium (20 g proteose peptone, 2 g yeast extract, 0.98 g MgSO_4_ ⋅ 7H_2_O, 0.35 g Na_2_HPO_4_ ⋅ 7H_2_O, 0.34 g KH_2_PO_4_, 0.02 g (NH4)_2_Fe(SO_4_)_2_ ⋅ 6H_2_O, 18 g glucose and 1,000 mL distilled water (DW) (All components in PYG were from HiMedia Laboratories., Mumbai, India) at 28°C and centrifuged at 1,520 × g for 5 min in fresh PYG medium before further testing.

### Anti-*Acanthamoeba* screening

The anti-*Acanthamoeba* activity of RAE was evaluated with slight modification [16, 17]. A final concentration of 8 mg/mL of RAE was initially performed against all species of tested *Acanthamoeba* trophozoites using the broth microdilution method in 96-well microtiter plates (SPL Life Sciences, Gyeonggi-do, Korea). One-hundred microliter of 16 mg/mL RAE were transferred to each well in triplicate, and 100 μL of inoculum (2 × 10^5^ cells/mL) was added. The plates were incubated at 28°C for 24 h. CHX (0.016 mg/mL; HiMedia Laboratories) and DMSO (2%) were used as positive and negative controls, respectively. The *Acanthamoeba* viability was calculated using the following equation:


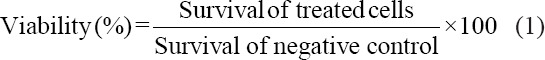


### Minimal inhibitory concentration (MIC) and minimal parasiticidal concentration (MPC)

After anti-*Acanthamoeba* screening, a MIC assay was performed as described previously by Mitsuwan *et al*. [16]. Briefly, 100 μL of RABE and RALE were serially 2-fold diluted to final concentrations of 16, 8, 4, 2, 1, and 0.5 mg/mL. Then, 100 μL of 2 × 10^5^ cells/mL of trophozoites were inoculated into each well of 96 well plate. CHX (0.001–0.128 mg/mL) and DMSO (2%) were used as positive and negative controls, respectively. After 24 h incubation, the *Acanthamoeba* viability was calculated using Equation (1). The MIC was defined as the lowest concentration that inhibited >90% of viable growth [16, 17]. The concentration at MIC and above MIC was further assessed using parasiticidal activity for 72 h incubation. Viability was counted and calculated using Equation (1). MPC was defined as the lowest concentration that inhibited >99.99% of viable growth after 72 h [16, 17].

### Scanning electron microscopic (SEM) study

The trophozoites of *A. triangularis* were treated with RABE and RALE (8 mg/mL) and CHX (0.008 mg/mL). After incubation, cells were collected by centrifugation at 1,520 × *g* for 5 min and re-suspended in phosphate buffer saline (PBS; Oxoid Holdings, Hampshire, UK). CHX and 2% DMSO were used as positive and negative controls, respectively. Samples were fixed with 2.5% glutaraldehyde overnight, dehydrated with a series of graded alcohols (20%, 40%, 60%, 80%, 90%, and 100% ethanol), mounted on aluminum stubs, and allowed to dry using a critical-point dryer. Samples were then coated with gold particles, and the morphology of *A. triangularis* trophozoites after the treatments was subsequently examined under SEM (SEM-Zeiss, Munich, Germany) [17].

### Minimal cytotoxicity concentration (MCC)

The MCC was performed as previously described by Chimplee *et al*. [18] and Sama-ae *et al*. [19]. The cytotoxicity of RABE and RALE was assessed using Vero cells (ECACC 84113001, RRID: CVCL 0059, Salisbury, UK) provided by Associate Professor Dr. Chuchard Punsawad, School of Medicine, Walailak University. Human epidermal keratinocyte (HaCaT) cells (CLS Cell Lines Service, Cytion cat no. 300493, DKFZ, Heidelberg, Germany) were also tested courtesy of Associate Professor Dr. Warangkana Chunglok and Dr. Nichaporn Wongsirojkul, School of Allied Health Sciences, Walailak University. Both cell lines were cultured in Dulbecco’s Modified Eagle’s medium (Merck KGaA, Darmstadt, Germany) containing 10% fetal bovine serum (Sigma Aldrich, St. Louis, USA) and 1% penicillin-streptomycin, incubated at 37°C in an atmosphere containing 5% CO_2_. Once the cells reached 90% confluence, they were detached with trypsin-ethylenediaminetetraacetic acid and incubated again for 5 min. Vero and HaCaT cells (1.5 × 10^4^ cell/mL) were seeded in 96-well plates and allowed to attach for 24 h before RABE and RALE (0.016–1 mg/mL) treatment. After 24 h, the cytotoxicity was assessed using the MTT (3-[4,5-dimethylthiazol-2-yl]-2,5 diphenyl tetrazolium bromide) assay. In brief, 100 μl of MTT reagent (0.5 mg/ml) was added to each well and the cultures were incubated for an additional 1 h. The MTT reagent was removed and replaced by 100% DMSO to ensure that solubilization was complete. Absorbance at 570 and 650 nm (reference wavelengths) was measured on a microplate reader. The survival percentage was calculated using the following equation:


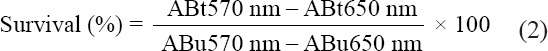


ABt and ABu denote the absorbance of treated and untreated cells (1% DMSO), respectively.

MCC was defined as the lowest concentration that inhibited <20% cell viability [20].

### Anti-adhesion activity

The anti-adhesion properties of RAE on *Acanthamoeba* were examined as previously described by Sangkanu *et al*. [21]. Trophozoites (4 × 10^5^ cells/mL) were seeded into a 96-well polystyrene microtiter plate containing RABE, RALE, CHX, and a multipurpose solution (MPS; Duna, Alcon Laboratories, TX, USA). The plates were incubated at 28°C without shaking for 24 h. After incubation, the medium and unbound trophozoites were discarded. The plates were washed once with 0.1 M PBS, air-dried, and stained with crystal violet (0.05%) for 30 min. The crystal violet was discarded, and the plates were washed with water and air-dried. An aliquot of DMSO was added to the well, and the absorbance was read at OD_570 nm_. Wells containing trophozoites in 2% DMSO were used as a control. The percentage of inhibition was calculated using the following equation:


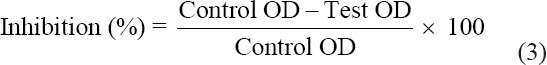


### Phytochemical analysis by gas chromatography-mass spectrometry (GC-MS)

The RABE and RALE (20 mg/mL) were diluted in ethanol (1:10). The solution was centrifuged for 10 min at 10,864 × *g*, 10°C. The solution was analyzed by GC-MS using Agilent Technology 7890 A (GC) equipped with a 5977A mass-selective detector (MS) (Agilent, California, USA). A VF-WAXms capillary column of dimensions 30 m × 250 × 0.25 μM was used with helium gas as the carrier at 30 m × 250 × 0.25 μM at a flow rate of 1 mL/min. The column temperature was initially programed at 60°C, which was increased to 10°C/min to 160°C/min, gradually increased to 2.5°C/min to 325°C/min, and held for 15 min. The mass spectra were collected at an ionization voltage of 70 eV over the range of m/z 35–500 in full-scan mode. Chemical constituents were identified by comparing their mass spectral data with those from the Wiley-Blackwell Library (Hoboken, NJ, USA).

### The protein three-dimensional (3D) structure prediction

This study focused on the *A. triangularis* beta-tubulin (At-β-tubulin) protein. The SWISS-MODEL service (Swiss Institute of Bioinformatics, Basel, Switzerland) was employed to predict the 3D structure of this protein because it has no crystal structure [22, 23]. The At-β-tubulin FASTA sequence (GenBank: AFI57878.1; National Library of Medicine, MD, USA) was used as the initial input, and ModRefiner was used to further improve the projected 3D model’s quality [24]. Finally, PROCHECK (EMBL-EBI, Cambridgeshire, UK) was used to assess the stereochemical quality of the protein structures [25].

### Ligand-binding pocket prediction

The ligand-binding pockets were predicted using DeepSite, a protein-binding pocket predictor based on deep neural networks [26]. The Protein Data Bank (PDB) format of the prepared protein structure was used as the input. The DeepSite prediction results were then used to set up the center of the grid box before the molecular docking process.

### Preparation of protein and ligand structures for molecular docking

The protein structure was dehydrated before molecular docking to reveal the amino acid residues and assigned a Kollman charge. Non-polar and polar hydrogens were merged and added to amino acid residues. Finally, partial charges and atom types were assigned to stabilized protein structures. The data were stored in the PDB, Partial Charge (Q), and Atom Type (T) PDBQT format.

To prepare the ligand, Gasteiger charges and polar hydrogens were added to the ligand structures, while non-polar hydrogens were merged. The ligand structures were also stored in PDBQT format. Grid maps depicting the system in the docking process were created using AutoGrid4 software version 4.2 (The Scripps Research Institute, CA, USA). The DeepSite prediction findings were used to determine the center of the grid box. The grid dimension was chosen to encompass the whole receptor assembly (60 × 60 × 60 Å), with a spacing of 0.375 Å. The AutoDock Auxiliary Tool (ADT) version 4.2 (The Scripps Research Institute, CA, USA) was used for molecular docking prediction [27, 28].

### Molecular docking of clionasterol and stigmasterol compounds to A. *triangularis-β*-tubulin

Molecular docking was performed using ADT version 4.2 [27, 28]. There were 50 GA runs in each docking stage (a maximum of 200 units). Therefore, a total energy evaluation of 2,500,000 units was required for each pier. Each docking had an average mutation rate of 0.02, cross-over rate of 0.80, and elitism value of 1.00 [29, 30]. The Lamarckian Genetic Algorithm was used to combine local (Solis and Wets algorithm) and global (Genetic Algorithm alone) search [31]. The predictable docking per complex was repeatedly performed to execute 10,000 separate docking for 5 times to ensure that the findings were correct. The inhibitor constant and the protein (P)-ligand (L) (At-β-tubulin-L) lowest binding energy (ΔG_bind_) were calculated using ADT version 4.2 [27, 28].

### Molecular dynamics simulation (MDS)

Schrödinger’s “Desmond v3.6 Program” (https://www.schrodinger.com) was used within a Linux framework to perform molecular dynamic simulations evaluating various At-β-tubulin complex structures to determine its binding consistency [31, 32]. The proposed framework was used to build a specified volume with an orthorhombic periodic bounding box shape divided into 10 using the pre-defined TIP3P aqueous method. To balance the electric charge inside the structure, suggested ions, such as 0+ and 0.15 M salt (Na^+^ and Cl^-^), were randomly distributed throughout the chemical solvent environment. Following the construction of the system’s solvency protein structures comprising agonist combinations, the protocol used to apply the force field constants OPLS3e included in the Desmond package (Schrödinger, NY, USA) was lowered and improved the system framework. Each Isothermal-Isobaric ensemble (NPT), which is a statistical assembly of how complexity is made use of the overall Nose–Hoover temperature combinations, and its isotropic method, was kept at a temperature of 300 K (26.85°C), an atmospheric pressure of 1 kPa (1.01325 bar), followed by 50 PS grabbing pauses with a 1.2 kcal/mol efficiency. Throughout the entire simulation, the fidelity of the MDS was evaluated using the simulation interaction diagram (SID) from the Desmond modules of the Schrödinger suite. The stability of At-β-tubulin-L complex combination was evaluated using data on At-β-tubulin-L interactions, intramolecular hydrogen bonds, solvent accessible surface area (SASA), radius of gyration (Rg), molecular surface area (MolSA), and polar surface area (PSA) [33, 34].

### Simulation trajectory analysis

Schrödinger’s maestro software, version 9.5 (Schrödinger, NY, USA), was used to produce the MDS images. The SID of the Desmond modules in the Schrödinger package was used to analyze the potential simulation scenario and evaluate the MDS accuracy. The stability of the At-β-tubulin-L complex framework was assessed using the root-mean-square deviation (RMSD), At-β-tubulin-L contacts, intramolecular hydrogen bonds, SASA, Rg, MolSA, and PSA values in accordance with the trajectory effectiveness.

### RMSD analysis

In MDS, the RMSD measures the average distances resulting from removing one molecule from a system over a pre-defined period in relation to a reference value [35–37]. In our 100-ns MDS analysis, the RMSD of protein structural atoms, such as C, foundation, sidechain, and bulkier components, was added after the RMSD of protein fit ligand atoms throughout each time frame, which was coordinated and evaluated against the benchmark time [34, 38, 39]. To determine the RMSD of an MDS with a period of x, apply the following equation (Equation 4).


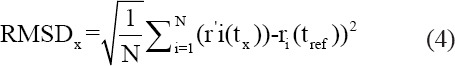


The letter r’ denotes the position of the selected atom in system X after superimposing the point of the standard system. Here, N denotes the number of atoms chosen, and t_ref_ denotes the reference time.

### Root mean square fluctuation (RMSF) analysis

The RMSF has mostly been used to identify and monitor regional changes in the translational structure of protein complexes [40, 41]. The continuity formula can be used to determine the RMSF frequency of an MDS of an amino acid with the number of residues I (Equation 5).


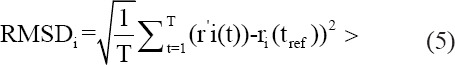


In this case, T denotes the trajectory time used to calculate the RMSF. With t_ref_ denotes the reference time, r_i_ denoting the location of residue i, and r’ denoting the position of atoms in residue i after superposition on the reference, the angle brackets indicate that the average of the square distance is calculated over a preference of atoms in the residue.

## Results

### Anti-*Acanthamoeba* activity

The anti-*Acanthamoeba* activity of RAE was screened for all *Acanthamoeba* species. RALE and RABE were evaluated for their active ingredients and MIC and MPC values (Table-1). The MIC values of extracts ranged from 8 to >8 mg/mL for trophozoites and cysts. *A. triangularis* trophozoites were inhibited at MIC = 8 mg/mL by both RALE and RABE, but *A. polyphaga* trophozoites were merely inhibited by RABE. The MPC values of RALE and RABE were 16 mg/mL when *A. triangularis* trophozoites were tested. CHX, a positive control, displayed the best anti-*Acanthamoeba* activity against trophozoites at MIC 0.008–0.016 mg/mL and cyst at MIC 0.0064–>0.0128 mg/mL (Table-1).

**Table-1:** MIC and MPC values of *Rhizophora apiculata* leaf and bark extracts against trophozoite and cystic forms of *Acanthamoeba* spp. and their MCC.

Extracts	MIC^a^ (MPC)^b^ concentration (mg/mL)								MCC^c^ (mg/mL)	
	
	*A. triangularis* WU 19001		*A. polyphaga* ATCC 30461		*A. castellanii* ATCC 50739		*A. castellanii* ATCC 30010		Vero	HaCaT
			
	Trop.	Cyst	Trop.	Cyst	Trop.	Cyst	Trop.	Cyst		
RALE	8 (16)	8 (>16)	>8 (nd)	>8 (nd)	>8 (nd)	>8 (nd)	>8 (nd)	>8 (nd)	0.25	0.25
RABE	8 (16)	8 (>16)	8 (>16)	>8 (nd)	>8 (nd)	>8 (nd)	>8 (nd)	>8 (nd)	0.25	0.25
CHX	0.008 (0.008)	0.064 (0.064)	0.016 (0.016)	>0.0128 (>0.0128)	0.016 (0.016)	0.032 (0.032)	0.008 (0.008)	>0.0128 (>0.128)	nd	nd

RALE=*Rhizophora apiculata* leaf extract, RABE=*Rhizophora apiculata* bark extract, Trop.: Trophozoites, nd: not detected,

a MIC=Minimal inhibitory concentration is defined as the lowest concentration that inhibits >90% of viable growth,

b MPC=Minimal parasiticidal concentration, defined as the lowest concentration that inhibited >99.99% of viable growth,

c MCC=Minimal cytotoxicity concentration is defined as the lowest concentration that inhibited <20% cell viability, ATCC=American Type Culture Collection

### Anti-adhesion activity

The efficacy of CHX in reducing the adhesion of extracts was evaluated at doses below the MIC. The RABE was the most effective in reducing the adhesion of *Acanthamoeba* trophozoites on a 96-well polystyrene microtiter plate at 4 mg/mL (Table-2). The percentages of anti-adhesion of 94.45 ± 0.22, 97.94 ± 1.86, 99.89 ± 0.02, and 98.57 ± 0.09 for *A. triangularis* WU 19001, *A. polyphaga* ATCC 30461, *A. castellanii* ATCC 50739 and *A. castellanii* ATCC 30010, respectively. RALE inhibited (more than 90%) adherent *Acanthamoeba* trophozoites of *A. triangularis* WU 19001, *A. polyphaga* ATCC 30461, and *A. castellanii* ATCC 30010. At 0.004 mg/mL CHX, the drug effectively prevented adhesion by more than 80%, but not *A. castellanii* ATCC 50739. MPS was effective against *A. triangularis* WU 19001 (84.92% ± 1.72%) but less effective against other *Acanthamoeba* strains.

**Table-2:** The inhibition of *Acanthamoeba* adherence by *Rhizophora apiculata* extracts.

Agent	Concentration (mg/mL)	Inhibition of adhesion (%)			

		*Acanthamoeba triangularis* WU 19001	*Acanthamoeba polyphaga* ATCC 30461	*Acanthamoeba castellanii* ATCC 50739	*Acanthamoeba castellanii* ATCC 30010
RALE	4	96.08 ± 0.83	94.69 ± 0.80	78.44 ± 5.84	94.62 ± 4.75
	2	88.44 ± 4.68	86.68 ± 3.84	58.88 ± 2.04	69.74 ± 4.08
RABE	4	94.45 ± 0.22	97.94 ± 1.86	99.89 ± 0.02	98.57 ± 0.09
	2	79.90 ± 7.33	85.27 ± 1.77	63.42 ± 5.77	66.64 ± 1.51
CHX	0.008	ND	85.39 ± 0.11	89.67 ± 1.34	ND
	0.004	87.64 ± 0.13	82.84 ± 0.77	71.36 ± 5.66	84.46 ± 0.75
	0.002	58.43 ± 5.92	ND	ND	81.31 ± 1.43
MPS	100%	84.92 ± 1.72	63.04 ± 11.97	63.35 ± 17.42	60.36 ± 13.87

RALE=*Rhizophora apiculata* leaf extract, RABE=*Rhizophora apiculata* bark extract, CHX=Chlorhexidine, ND=Not detected, MPS=Multipurpose solution, ATCC=American Type Culture Collection

### SEM study

**Figure-2:**
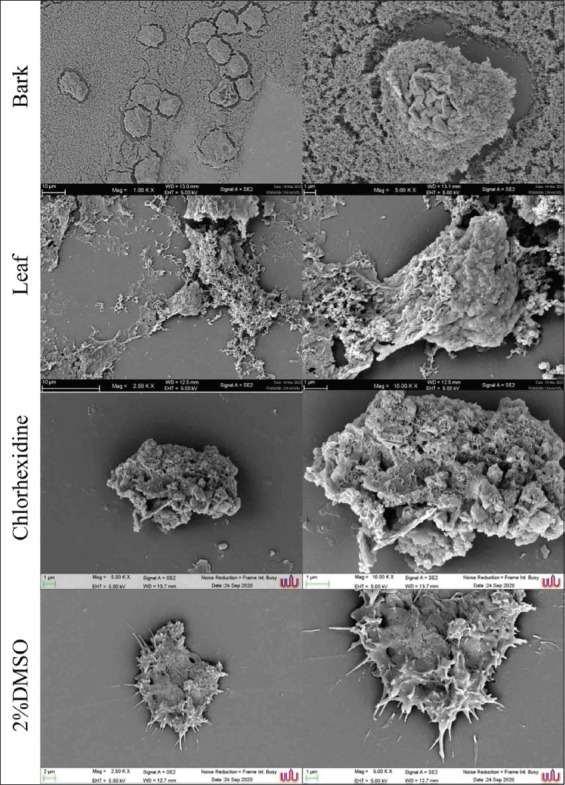
Scanning micrographs of *Acanthamoeba triangularis* trophozoites after treatment with bark and leaf extracts of *Rhizophora apiculata* and chlorhexidine at minimum inhibitory concentrations (8 and 0.008 mg/mL) for 24 h. The control trophozoite was treated with 2% dimethyl sulfoxide. The horizontal panel indicates the increase in the magnification from left to right.

SEM was used to study the adherent ability of RAE on *A. triangularis*. Figure-2 shows the morphological changes of *A. triangularis* trophozoites incubated with MICs of RALE, RABE, and CHX (positive control). Our finding shows the presence of pores and the loss or damage of acanthopodia was clearly shown after treatment with those extracts when compared to acanthopodia on the surface of *A. triangularis* in the negative control (2% DMSO).

### Cytotoxicity

The RALE and RABE demonstrated low cytotoxicity toward Vero and HaCaT cell lines, with greater than 80% cell viability at an MCC of 0.25 mg/mL (Table-1).

### GC-MS analysis

This study determined the bioactive compounds present in the RAE using GC-MS. The total molecular formulas and percentages of the active compounds are presented in Table-3. The major compounds found in the RABE were α-amyrin (15.73%), vitamin E (11.36%), γ-sitosterol (clionasterol) (8.61%), germanicol (6.43%), β-amyrin (5.62%), phytyl acetate (2.01%), hexadecanoic acid (palmitic acid) (1.71%), and S-methyl methanethiosulfonate (1.53%). While another 11 major compounds found in the RALE were lupeol (26.53%), β-amyrin (10.07%), DL-α-tocopherol (8.51%), squalene (6.89%), γ.-sitosterol (6.42%), phytyl acetate (2.49%), phytol (2.02%), stigmasterol (1.3%), neophytadiene (1.2%), hexadecanoic acid (palmitic acid) (1.2%), and lupeol trifluoroacetate (1.13%).

**Table-3:** Chemical profiles based on GC-MS analysis of bark and leaf *Rhizophora apiculata* extracts.

No.	Compound Name	Formula	% Total	

			RABE	RALE
1.	S-Methyl methanethiosulfonate	C_2_H_6_O_2_S_2_	1.53	0.59
2.	β-damascone	C_13_H_20_O	-	0.09
3.	6S-2,3,8,8-Tetramethyltricyclo[5.2.2.0 (1,6)]undec-2-ene	C_15_H_24_	-	0.13
4.	24-Noroleana-3,12-diene	C_2_9H_46_	-	0.14
5.	Valerena-4,7 (11)-diene	C_15_H_24_	-	0.08
6.	Cyclohexasiloxane, dodecamethyl-	C_12_H_36_O_6_Si_6_	0.54	-
7.	Megastigmatrienone2	C_13_H_18_O	0.42	0.27
8.	Ethyl alpha-d-glucopyranoside	C_8_H_16_O_6_	-	0.83
9.	Corchoionol C	C_13_H_20_O_2_	-	0.18
10.	Pluchidiol	C_13_H_20_O_2_	-	0.66
11.	Neophytadiene	C_20_H_38_	-	1.2
12.	3,7,11,15-Tetramethylhexadecyl acetate	C_22_H_44_O_2_	-	0.13
13.	Phytyl acetate	C_22_H_42_O_2_	2.01	2.49
14.	Hexadecanoic acid (Palmitic acid)	C_16_H_32_O_2_	1.71	1.2
15.	Hexadecanoic acid, ethyl ester	C_18_H_36_O_2_	0.39	0.21
16.	Phytol	C_20_H_40_O	-	2.02
17.	Oleic acid	C_18_H_34_O_2_	-	0.32
18.	Linoleic acid ethyl ester	C_20_H_36_O_2_	0.11	0.04
19.	Ethyl linolenate	C_20_H_34_O_2_	-	0.25
20.	ent-Atisan-16.alpha.-ol	C_20_H_34_O	-	0.27
21.	Palmitate de 1,3-dihydroxy-2-propanyle	C_1_9H_38_O_4_	-	0.51
22.	Tricosanal	C_23_H_46_O	-	0.23
23.	Ethyl iso-allocholate	C_26_H_44_O_5_	-	0.45
24.	1,6,10,14,18,22-Tetracosahexaen-3-ol, 2,6,10,15,19,23-hexamethyl-, (all-E)-	C_30_H_50_O	-	0.15
25.	Squalene	C_30_H_50_	-	6.89
26.	Olean-13 (18)-ene	C_30_H_50_	-	0.08
27.	9,19-Cyclolanost-24-en-3-ol, acetate, (3.beta.)-	C_32_H_52_O_2_	-	0.31
28.	supraene	C_30_H_50_	0.42	-
29.	α-Tocospiro A	C_2_9H_50_O_4_	0.27	-
30.	DL-α-Tocopherol	C_2_9H_50_O_2_	-	8.51
31.	Germanicol	C_30_H_50_O	0.61	0.65
32.	2H-1-Benzopyran-6-ol, 3,4-dihydro-2,7,8-trimethyl-2-(4,8,12-trimethyltridecyl)-	C_28_H_48_O_2_	0.28	0.24
33.	Ursa-9 (11),12-dien-3-ol	C_30_H_48_O	-	0.44
34.	Lupeol, trifluoroacetate	C_32_H_4_9F_3_O_2_	-	1.13
35.	24-Norursa-3,12-diene	C_2_9H_46_	0.9	-
36.	β-Sitosterol acetate	C_31_H_52_O_2_	0.82	-
37.	β -Sitosterol, propionate	C_32_H_54_O_2_	-	0.34
38.	D: A-friedo-olean-6-ene	C_30_H_50_	-	0.27
39.	Tricyclo[5.4.3.0 (1,8)]tetradecan-3-ol-9-one, 4-ethenyl-6-(2-hydroxyacetoxy)-2,4,7,14-tetramethyl-	C_22_H_34_O_5_	-	0.78
40.	Vitamin E	C_2_9H_50_O_2_	11.36	-
41.	Campesterol	C_28_H_48_O	0.54	0.63
42.	γ-Sitosterol (Clionasterol)	C_2_9H_50_O	8.61	6.42
43.	Germanicol	C_30_H_50_O	6.43	-
44.	β-Amyrin	C_30_H_50_O	5.62	10.07
45.	α-Amyrin	C_30_H_50_O	15.73	-
46.	Lupeol	C_30_H_50_O	0.62	26.53
47.	Stigmasterol	C_2_9H_48_O	-	1.3
48.	Lup-20 (29)-en-3-one	C_30_H_48_O	-	0.35
49.	Androst-5-en-17-ol, 4,4-dimethyl-	C_21_H_34_O	-	0.26
50.	24 methyl-23-dehydro-cycloartanol	C_31_H_52_O	-	0.3
51.	Lupan-3-ol, acetate	C_32_H_54_O_2_	-	0.58
52.	3-β-Acetoxy-11-oxoursan-12-ene	C_32_H_50_O_3_	-	0.35
53.	Betulinaldehyde	C_30_H_48_O_2_	-	0.29

RALE=*Rhizophora apiculata* leaf extract, RABE=*Rhizophora apiculata* bark extract, Match factor more than 70%

### The protein three-dimensional (3D) structure prediction

**Figure-3:**
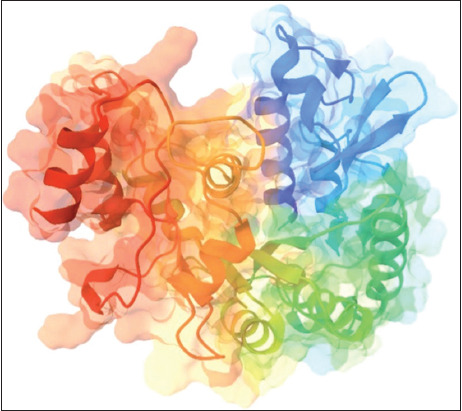
Predicted three-dimensional (3D) structures of *Acanthamoeba triangularis* on β-tubulin.

The SWISS-MODEL service creates a 3D structural model of At-β-tubulin using the tubulin β-4B chain as a template. Figure-3 illustrates the 3D structural model of At-β-tubulin. The sequence identity percentage with the template was 80.63%. The projected structure’s QMEANDisCo Global was 0.75 ± 0.05. The Ramachandran plot of the At-β-tubulin model detected 94.2% of the residues in the most favored regions and 0.00% in the disallowed regions, indicating generated protein structures with good stereochemical quality [42].

### Molecular docking

AutoDock 4 was used for the molecular docking of major chemicals in RABE and RALE to At-β-tubulin proteins (Table-4). Clionasterol and stigmasterol displayed good binding capability to the ligand binding of At-β-tubulin, with ΔG_bind_ of 11.37 and 11.64 kcal/mol and K_i_ of 4.63 and 2.92 nM, respectively. In drug control, CHX had a ΔG_bind_ of −10.53 kcal/mol and an K_i_ of 19.19 nM. Benzimidazole had ΔG_bind_ of −4.44 and K_i_ of 557.65 μM.

**Table-4:** Binding affinity and inhibitory constant prediction of *Rhizophora apiculata* compounds against β-tubulin protein of *Acanthamoeba triangularis.*

Compound name	PubChem CID	Binding affinity (ΔG_bind_, kcal/mol)	Inhibitory constant (K_i_)
RABE			
Hexadecanoic acid (Palmitic acid)	985	−4.37	626.15 uM
Vitamin E	14985	−10.19	34.11 nM
S-Methyl methanethiosulfonate	18064	−3.84	1.53 mM
β-Amyrin	73145	−10.93	9.77 nM
α-Amyrin	73170	−11.25	5.7 nM
Germanicol	122857	−11.14	6.85 nM
Clionasterol	457801	−11.37	4.63 nM
Phytyl acetate	637195	−6.88	9.02 uM
RALE			
Hexadecanoic acid (Palmitic acid)	985	−4.37	626.15 uM
DL-α-Tocopherol	2116	−9.72	75.57 nM
Neophytadiene	10446	−6.82	10.1 uM
β-Amyrin	73145	−10.93	9.77 nM
Lupeol	259846	−11.33	4.93 nM
Clionasterol	457801	−11.37	4.63 nM
Phytyl acetate	637195	−6.88	9.02 uM
Squalene	638072	−8.88	307.56 nM
Phytol	5280435	−6.4	20.2 uM
Stigmasterol	5280794	−11.64	2.92 nM
Lupeol, trifluoroacetate	91704083	−11.39	4.49 nM
Drug			
Benzimidazole	5798	−4.44	557.65 uM
Chlorhexidine	9552079	−10.53	19.19 nM

RALE=*Rhizophora apiculata* leaf extract, RABE=*Rhizophora apiculata* bark extract

**Figure-4:**
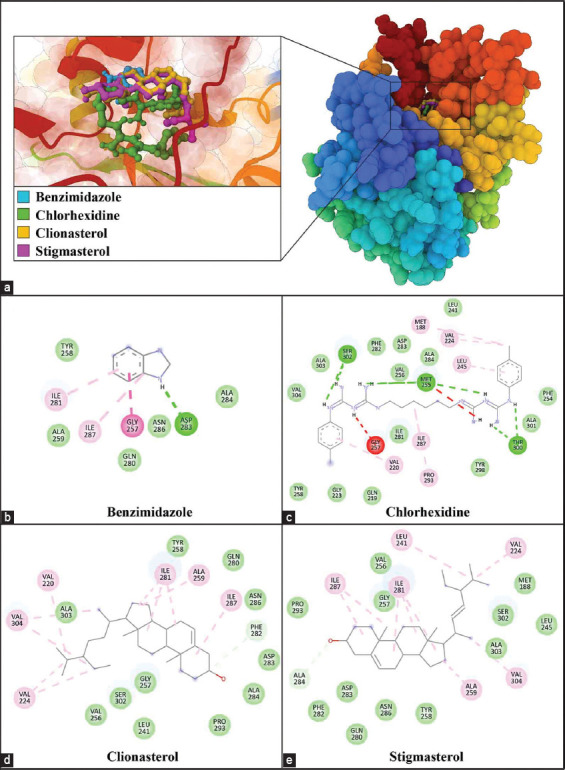
The figure illustrates the binding site and two-dimensional interaction diagrams depicting the interactions of ligands with *Acanthamoeba triangularis*-β-tubulin. In panel (a), the binding site is presented, highlighting the spatial arrangement of benzimidazole (depicted in blue), chlorhexidine (depicted in green), clionasterol (depicted in yellow), and stigmasterol (depicted in pink) within the *A. triangularis*-β-tubulin structure. Panels (b-e) provide detailed two-dimensional interaction diagrams for benzimidazole, chlorhexidine, clionasterol, and stigmasterol, respectively, elucidating the specific interactions each ligand forms with *A. triangularis* β-tubulin at the molecular level.

Analysis of molecular interactions revealed that benzimidazole establishes hydrogen bonds with the residue Asp283 of At-β-tubulin, in addition to engaging in van der Waals interactions with Tyr258, Ala259, Gln280, Ala284, and Asn286. Furthermore, benzimidazole forms interactions with Ile281 and Ile287 (Alkyl/Pi-Alkyl) and Gly257 (Pi-Pi T shaped/Amide-Pi Stacked) residues of At-β-tubulin. CHX, on the other hand, establishes hydrogen bonds with Thr300, Ser302, and Met255 residues of At-β-tubulin, accompanied by van der Waals interactions with Gln219, Gly223, Leu241, Phe254, Val256, Tyr258, Ile281, Phe282, Asp283, Ala284, Tyr298, Ala301, Ala303, and Val304 residues. In addition, CHX forms Alkyl/Pi-Alkyl interactions with the Met188, Val220, Val224, Leu245, Ile287, and Pro293 residues of At-β-tubulin. Clionasterol establishes hydrogen bonds with the Phe282 residues of At-β-tubulin, along with van der Waals interactions involving Leu241, Val256, Gly257, Tyr258, Gln280, Asp283, Ala284, Asn286, Pro293, Ser302, and Ala303 residues. Moreover, clionasterol interacts with the alkyl/pi-alkyl residues Val220, Val224, Ala259, Ile281, Ile287, and Val304 of At-β-tubulin. Finally, stigmasterol forms hydrogen bonds with Ala284 residues of At-β-tubulin, accompanied by van der Waals interactions with Met188, Leu245, Val256, Gly257, Tyr258, Gln280, Phe282, Asp283, Asn286, Pro293, Ser302, and Ala303 residues. In addition, stigmasterol participates in Alkyl/Pi-Alkyl interactions with the Val224, Leu241, Ala259, Ile281, Ile287, and Val304 residues of At-β-tubulin (Figure-4).

### RMSD analysis

The MDS has been used to explore the system at an atomistic level and to determine the conformational strength of atoms and molecules. MDS is a precise and distinctive method for examining the stability of ligands in certain proteins. The complex structure of the selected chemicals was examined in this instance using a 100 ns MDS. This was performed to test how well they could bind to the protein and the cavity that houses the protein’s active site. The RMSD, RMSF, SASA, MolSA, PSA, intramolecular hydrogen bonds (Intra HB), and At-β-tubulin-L contact analysis were used to explain the outcomes of MDS.

**Figure-5:**
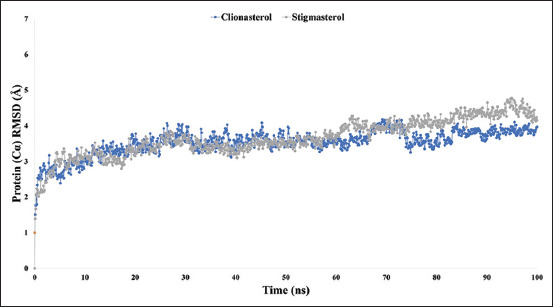
Graphs showing the root-mean-square deviation values for the two ligand molecules in complex with the protein beta-tubulin (*Acanthamoeba triangularis*-β-tubulin) in 100 ns molecular dynamics simulation assessments, where the selected two ligands, the compound clionasterol, and stigmasterol are associated with the protein are exhibited by blue and gray colors.

The average distance induced by a particular atom’s dislocation over a pre-determined period can be calculated using At-β-tubulin-L complex system’s RMSD. To calculate the difference between two observed and estimated values, the square root of the mean of the squared errors is typically used. A value outside the permitted range denotes a considerable conformational shift in the protein. As shown in Figure-5, the RMSD of the At-β-tubulin protein was compared with the complex structure of the drug candidate compounds clionasterol (blue) and stigmasterol (gray) to observe changes in the order. The RMSD values for the two compounds ranged from 1.0 to 4, with slight fluctuations that were perfectly acceptable.

### RMSF analysis

The RMSF enables one to compute the average change observed over a significant number of atoms and evaluate the displacement of a specific atom relative to the reference structure, which is essential for observing local protein alterations. Similar to the RMSD, this numerical computation is significant for characterizing proteins.

**Figure-6:**
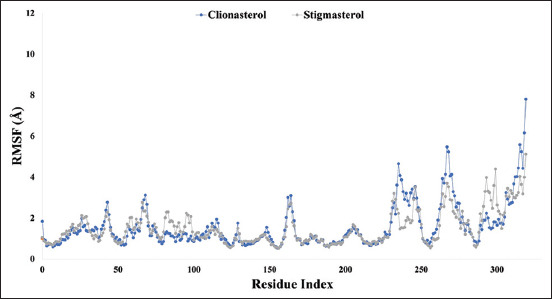
Graphs showing the root mean square fluctuation values for the two ligand molecules in complex with the targeted protein beta-tubulin (*Acanthamoeba triangularis*-β-tubulin) in 100 ns molecular dynamics simulation assessments, where the selected two ligands, the compound clionasterol, and stigmasterol in associated with the protein are exhibited by blue and gray color.

To analyze the change in protein structural flexibility generated by the attachment of the chosen ligand compounds to a particular residual position, the RMSF values of the experimental drug candidate compounds clionasterol (blue) and stigmasterol (gray) in complex with the At-β-tubulin protein were calculated, as shown in Figure-6. Analysis of the RMSF graph revealed fluctuations between 235 and 319 residues for clionasterol with a maximum range of 7.7 A, indicating less stability of the compounds, whereas stigmasterol showed low fluctuations, suggesting stability of the system.

### The radius of gyration (Rg) analysis

**Figure-7:**
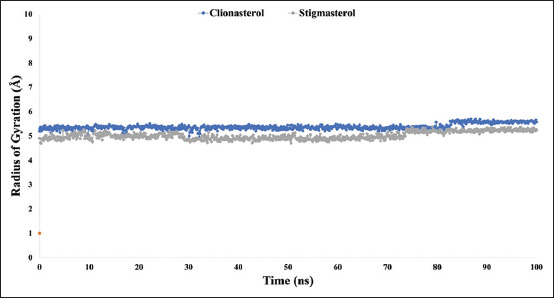
The graphs show the radius of gyration values for the ligand molecules in complex with the targeted protein β-tubulin (*Acanthamoeba triangularis*-β-tubulin) in 100 ns molecular dynamics simulation assessments, where the selected two ligands, the compound clionasterol and stigmasterol, are associated with the protein are exhibited by blue and gray color.

The arrangement of its atoms around its axis can be used to define the Rg of At-β-tubulin-L interaction complex. One of the most crucial metrics to consider when predicting how a macromolecule’s structural functioning of this large molecule is the computation of Rg because it reveals changes in complex compactness throughout the course of the simulation. As a result, throughout the course of a 100-ns simulation, Figure-7 shows the stability of the therapeutic candidate compounds clionasterol (blue) and stigmasterol (gray) in interaction with the target protein was examined in terms of Rg. The median Rg values for the potential drugs and the computed binding constants for the ligands clionasterol (blue) and stigmasterol (gray) with the At-β-tubulin protein were 5.2 and 4.8, respectively. This suggests that there were no significant changes in the protein’s binding site structure when the ligand was bound.

### Analysis of SASA, MolSA, and PSA

**Figure-8:**
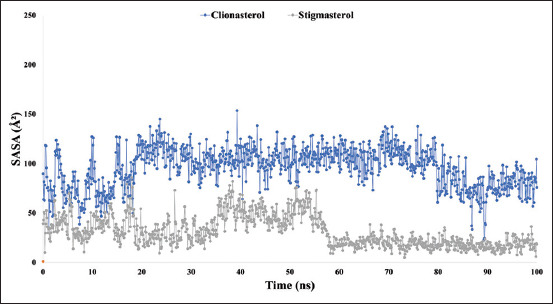
The graphs show the solvent accessible surface area values for the two ligand molecules in complex with the targeted protein β-tubulin (*Acanthamoeba triangularis*-β-tubulin) in 100 ns molecular dynamics simulation assessments, where the selected two ligands, the compound clionasterol, and stigmasterol in associated with the protein are exhibited by blue and gray colors.

The SASA measures a biological macromolecule’s organization and function. Proteins frequently have active sites and/or ligand-binding sites that can be exploited to better understand their solvent-like characteristics (hydrophilic or hydrophobic) and At-β-tubulin-L interactions. The SASA value for the compound structure ranged from 10 to 120 A^2^, indicating that the chosen ligand compounds were present at high concentrations where amino acid residues were present in complex systems. Figure 8 shows the computed and displayed SASA values for the therapeutic candidate compounds clionasterol (blue) and stigmasterol (gray) with the targeted At-β-tubulin protein.

**Figure-9:**
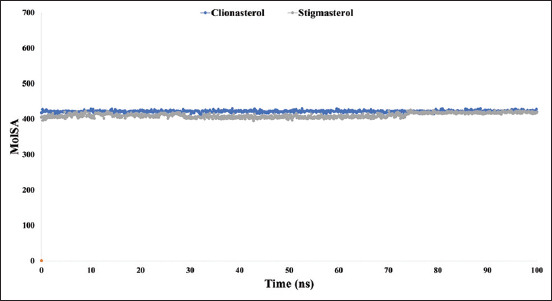
The graphs show the molecular surface area values for the two ligand molecules in complex with the targeted protein beta-tubulin (*Acanthamoeba triangularis*-β-tubulin) in 100 ns molecular dynamics simulation assessments, where the selected two ligands, clionasterol and stigmasterol, in associated with the protein are exhibited by blue and gray colors.

When the probe radius is adjusted to 1.4, the MolSA is equal to the van der Waals surface area. The drug candidate compounds clionasterol (blue) and stigmasterol (gray) with the At-β-tubulin protein in our *in-silico* analysis possessed the typical van der Waals surface areas with average scores of 406 and 404, respectively, as illustrated in Figure-9.

**Figure-10:**
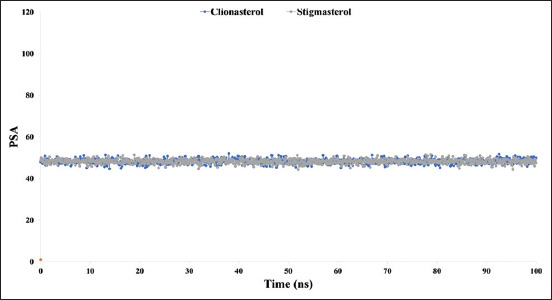
The graphs show the polar surface area values for the two ligand molecules in complex with the targeted protein beta-tubulin (*Acanthamoeba triangularis*-β-tubulin) in 100 ns molecular dynamics simulation assessments. The selected ligands, clionasterol and stigmasterol associated with the protein, are exhibited by blue and gray colors.

In addition, only oxygen and nitrogen atoms contribute to a structure’s PSA. With the At-β-tubulin protein, clionasterol (blue) and stigmasterol (grey) displayed a high PSA value of 47 for both compounds (Figure-10).

### Evaluation of intramolecular bonds

**Figure-11:**
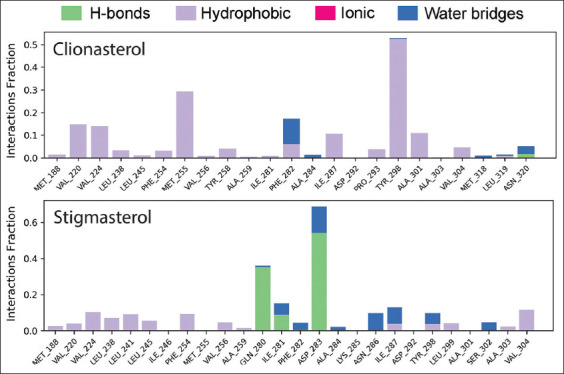
During the 100-ns simulation, the interactions with intramolecular bonds between proteins and ligands were analyzed (stacked bar charts). The figure represents the interaction of the ligands clionasterol and stigmasterol with the targeted protein beta-tubulin (*Acanthamoeba triangularis*-β-tubulin).

The protein complexes with the designated ligands and their intermolecular interactions were examined using the SID over the length of the 100-ns simulation. Figure-11 describes and illustrates these interactions (or “contacts”) between the drug candidates clionasterol (blue) and stigmasterol (gray) and the targeted At-β-tubulin protein. Each substance produces several connections through hydrogen, hydrophobic, ionic, and water-bridge bonding. It kept them until the simulation was over, making it easier to create strong interactions with the desired protein.

## Discussion

In this study, ethanolic crude extracts derived from the RALE and RABE exhibited potent cytotoxicity against *Acanthamoeba*. The MIC of 8 mg/mL, which reduced the survival of 90% of tested *Acanthamoeba* strains; mangrove extracts showed significant effects on *A. triangularis* (RABE and RALE) and *A. polyphaga* (RABE). To the best of our knowledge, this is the first study to evaluate the anti-*Acanthamoeba* activity of RABE and RALE against *Acanthamoeba* strains and compare them with other plant parts. To support this, Chandrasekaran *et al*. [43] have reported that the bark and leaf extracts of *Rhizophora* species and their active compounds have several biological activities, including anti-bacterial activity against methicillin-resistant *Staphylococcus aureus* with MIC of 0.125–4 mg/mL (methanolic extracts) and 1–16 mg/mL (aqueous extracts), and anti-fungal activity against *Trichophyton mentagrophytes*, *Microsporum canis*, *Trichophyton rubrum*, and *Epidermophyton floccosum* with MIC 0.25–2 mg/mL [17]. *Rhizophora racemosa* G.F.W. Mey. stem bark ethanolic extracts showed the best MIC 0.18–0.30 mg/mL against these fungi and anti-oxidant activity [17]. In addition, bark mangrove extracts exhibited ethanol-induced hepatocellular protection at 100–400 mg extract/kg body weight [44]. The anti-cancer effect of the stems of *R. mucronata* was evaluated in terms of cytotoxicity against various types of cancer, such as lung, colon, and breast cancer cell lines [45, 46]. It was found that the anti-cancer effect of the methanol extract from the stems of *R. mucronata* was greater than that of the leaf extract [45]. Overall, our anti-*Acanthamoeba* activity of RAE with MIC values was consistent with those of previous studies reported on anti-microbial activities and promising cytotoxic effects [17, 43-46].

The ability of *Acanthamoeba* to bind to epithelial cells is the basis of infection [47]. Tubulin is a microtubular monomer that plays a pivotal role in chromosomal segregation, organelle movement, and cellular motility [18]. Tubulin involves several cellular activities, such as mitosis, cytoskeleton movement and preservation of cell shape [45, 46]. The anti-adhesion property of RABE and RALE showed loss of acanthopodia, as observed by SEM (Figure-2), and reduced the adherence (about 80%–90%) of *A. triangularis* on the experimental 96 well plates (Table-2). The results suggest that acanthopodia of *Acanthamoeba* and surface adhesion ability are directly correlated with pathogenicity [47]. Hence, both RABE and RALE have the potential to reduce the surface adhesion of *A. triangularis* and *A. polyphaga*, which ultimately leads to a decrease in *Acanthamoeba* infections.

In the literature, >90% of the treatments for *Acanthamoeba* infections use various single or combinations of drugs such as amphotericin B, rifampin, trimethoprim-sulfamethoxazole, ketoconazole, fluconazole, sulfadiazine, miltefosine, albendazole, nitroxoline, and CHX [48, 49]. Nevertheless, current treatment for *Acanthamoeba* infections lasts up to a year and causes recurrent infection in approximately 10% of cases [49]. In addition, tubulin-targeting drugs are considered an alternative treatment for *Acanthamoeba* infections, but they are reported to cause resistance [11]. Although there were no novel findings of chemicals from GC/MS data of RABE and RALE, the extracts contained different potential chemical compounds (Table-3) that require more studies related to mechanisms of action and therapeutic approaches in the future.

All phytochemical compounds from GC-MS analysis were used as candidates to study their binding ability with β-tubulin protein of *Acanthamoeba*. It is known that a GTPase domain found in the self-polymerizing FtsZ protein family is shared between tubulin monomers, which is crucial for protozoan cell division [50]. Thus, tubulin has been used as a target for antiprotozoal agents [51]. In the present study, we utilized computational analysis to forecast the prospective impacts of the chemical components generated from RAE on β-tubulin protein. Molecular dynamic modeling can be used to ensure the compactness of At-β-tubulin-L complexes when a protein is in a complex with ligands. It can also determine how stable the complexes in specific environments, particularly in the human body. Examining the RMSD value enables the determination of the stability of chemical substances. In this process, the RSMF value is also used to calculate the At-β-tubulin-L complex’s evolution rate. In this study, we ran a 100-ns MDS with the relevant physiological and physicochemical parameters using the Schrödinger package software (Desmond Application) [25, 27–29]. This simulation tool’s trajectory has been used for various purposes. The RMSD and RMSF are a couple of examples of metrics that have been used to examine the Rg, hydrogen bond number, SASA, MolSA, and PSA. Higher RMSF values suggest that the At-β-tubulin-L complex is less compact in complex systems, whereas lower RMSD values suggest that compounds are more stable [32, 36, 52].

The targeted At-β-tubulin protein demonstrated potent RMSD and RMSF values with the two-drug candidates of clionasterol and stigmasterol as phytochemical compounds from GC-MS analysis, indicating promising therapeutic agents (Figures-5 and 6). When Rg is calculated, the stability of the protein structure is evaluated by measuring the center of mass from its C and N terminals [53–55]. This information provides a better understanding of how proteins fold. Regarding the targeted At-β-tubulin protein, a lower Rg value denotes a high degree of compactness, whereas a greater value denotes compound dissociation from the protein. With the targeted At-β-tubulin protein, the therapeutic candidate compounds clionasterol and stigmasterol exhibited reduced Rg values in our study (Figure-7). The simulation trajectories were also used to determine how the size of the drug-like molecules varied periodically. In addition, the At-β-tubulin-L SASA and its complex structure become less stable as the SASA value increases, but the lower the SASA value, the more tightly packed the water molecules and amino acid residues are [34]. The therapeutic candidate compounds showed decreased SASA values with the At-β-tubulin protein, according to the SASA result from the MDS trajectory (Figure-8). Our results showed that the drug candidate compounds had potential value, as shown in the graph of the MolSA and PSA validation (Figures-9 and 10). In addition, during a 100-ns simulation, the SID was employed to investigate the intermolecular interactions between proteins in complex with specific ligands. All ligands were shown to generate multiple connections through hydrogen bonds, hydrophobic bonds, ionic bonds, and water bridge bonds during the simulation and to maintain these connections until the simulation was complete. This facilitated the stable binding of the target protein (Figure-11).

Interestingly, this is the first study in which RAE was investigated to test against *Acanthamoeba* infections using *in-silico* analysis. In recent years, *in-silico* studies have been incorporated as an additional tool to validate promising phytochemical compounds derived from medicinal plants, especially for the benefit of neglected tropical diseases like acanthamoebiasis [19]. Overall, our study demonstrated that the predictive preliminary data from this *in-silico* study might play a role as a potential therapeutic agent for *Acanthamoeba* infections in the future. However, more comprehensive studies (e.g., nanoformulation) are required for the validation of this study’s results.

## Conclusion

To the best of our knowledge, this is the first report to investigate the adherent inhibitory of ethanolic RAE on tubulin interactions *in-silico*. Our findings revealed that RABE and RALE exerted the highest inhibitory effects on anti-adherence activity, especially against *A. triangularis and A. polyphaga*, and showed low toxicity in mammalian cells. The *in-silic*o study further investigated the binding capacities of clionasterol and stigmasterol, with β-tubulin. Based on our preliminary results, it is able to develop possible therapeutic agents as a potentially sustainable one-health approach for *Acanthamoeba* infections found in humans and animals.

## Data Availability

All data generated during the study are included in the manuscript.

## Authors’ Contributions

VN: Supervised the study. VN, SC, and SS: Conceptualized study and drafted and revised the manuscript. SC, SS: Produced the figures and tables and performed an experiment of anti-*Acanthamoeba* effects *in-vitro*. SC, SS, WM, JC, RB, IS, and DAK: Performed statistical analysis. IS and DAK: Performed *in-silico* study, and produced *in-silico* images. PB, MNH, HAT, CSS, PW, MLP, MN, RB, SSS, and AKP: Interpreted the results and drafted, edited, and revised the manuscript. All authors have read and approved the final manuscript.
